# PET CT Imaging with FDG in the Therapeutical Management of Locally Advanced Cervical Cancer Diagnosed in a 43-Year-Old Patient: Case Report and Review of the Literature

**DOI:** 10.3390/biomedicines13010083

**Published:** 2025-01-01

**Authors:** Ottó Molnar, Simona Mihuțiu, Oreste Mihai Straciuc, Alexandra Vesa, Liviu Lazar

**Affiliations:** 1 Doctoral Studies Department, Biomedical Science, 410087 Oradea, Romania; straciuc.mihai@gmail.com (O.M.S.); lazarlv@yahoo.com (L.L.); 2 Department of Medicine-Psycho-Neuroscience and Recovery, Faculty of Medicine and Pharmacy, 410073 Oradea, Romania; 3 Oncology Department, Pelican Hospital, 410469 Oradea, Romania; 4 Centrul PET/CT Pozitron Diagnosztika, 410035 Oradea, Romania; 5 Department of Morphological Sciences, Faculty of Medicine and Pharamacy, 410073 Oradea, Romania; alexvesa92@yahoo.com; 6 Băile Felix Medical Rehabilitation Hospital, 417500 Băile Felix, Romania

**Keywords:** FDG-PET/CT, SUVmax, LACC, fluorodeoxyglucose F-18, cervical cancer, diagnosis, radiotherapeutical management, brachytherapy, Ir-192, histopathological examination

## Abstract

**Background:** Cervical cancer is the most important cancer type found in women throughout the world. Numerous research studies are being performed to investigate the effectiveness of different strategies for the imaging and treatment of locally advanced cervical cancer, which are showing favorable outcomes. Brachytherapy is characterized by the application of very high radiation doses to target tumor cells with the least exposure to normal tissues. **Methods:** In the present case study, we report a 43-year-old female patient suffering from cervical cancer belonging to urban origin, with no personal pathological history, who presented herself to the gynecology department of the Bihor County Emergency Clinical hospital with vaginal bleeding. The histopathological examination of the cervix showed squamous cell carcinoma. The treatment was performed with neoadjuvant chemotherapy and concurrent chemoradiotherapy. **Results:** According to the clinical and histopathological examination, a diagnosis of non-keratinizing squamous carcinoma of the uterine cervix at FIGO stage III C1 was established. Radio-chemotherapy was performed, as well as periodic imaging assessments with a CT of the chest, pelvis, and abdomen, without local and distant relapse. FDG PET imaging was performed for the management and follow-up of cervical cancer by retrieving the SUVmax value. **Conclusions:** The post-therapeutic complications are represented by the vaginal stenosis installed 6 months after the end of the radiotherapeutic treatment.

## 1. Introduction

Cervical cancer is considered as the fourth-prevalent type of cancer in females throughout the world [1]. In patients at FIGO stages Ib, IIa, IIb, III, and IVa, recurrence rates range from 10 to 74%, and about 80% of cervical cancer (CC) recurrences happen within two years after primary treatment [2,3]. While dealing with cervical cancer, the regular metastatic sites are extra-pelvic nodes, liver, lungs, and the bones. CCs are mostly detected at later stages of onset, and many cancer patients do not receive proper care consistent with international standards [4]. The accurate staging and clinical evaluation of various prognostic factors are very essential prior to treatment for predicting a multidisciplinary method for therapy. Treatment options always appear challenging because of tumor size, its location, and an increased risk of the impairment of abnormal tissues to the normal ones. Almost 70 percent of patients with recurrent cervical cancer are reported to receive radiotherapy as the standard treatment strategy [5].

PET/CT imaging with innovative radiopharmaceuticals has the potential to significantly affect locally advanced cervical cancer (LACC) treatment by serving as markers for targeted drug delivery at the tumor microenvironment. In the early assessment of disease extent in LACC, 18F-fluorodeoxyglucose (FDG) PET/CT has become crucially important. FDG PET/CT is a valuable imaging technique for monitoring many human solid tumors, and it is currently required for staging, re-staging, and assessing therapeutic response in cervical cancer patients. This imaging technique combines 2-fluoro-2-deoxy-D-glucose (FDG), a structural glucose analogue, with the positron emitter fluorine-18 (18F-FDG), resulting in a noninvasive functional molecular diagnostic modality. 18F-FDG-PET outperforms other imaging techniques for managing solid tumors in people, monitoring lymph node status, and detecting distant metastases [6]. It is particularly useful for evaluating therapeutic response three months after completing concurrent chemoradiotherapy (CCRT), expecting long-term survival, and the detection of disease recurrence. Integrated PET/CT imaging works by combining PET images with anatomical CT images, proving to be more accurate than high-resolution CT only, particularly for regional lymph node detection and the extra-pelvic disease extension [7,8]. In the era of image-guided adaptive radiotherapy, accurately delineating disease areas is essential to prevent the unnecessary irradiation of normal tissue. FDG-PET provides additional information that allows for the modification of radiation treatment volumes, enabling the safe delivery of high and precise doses to FDG-positive lymph nodes. There is a scientific basis for the combination of innovative non-cytotoxic medicines with CRT, and medications that target specific biochemical pathways are currently in clinical trials. The maximum standardized uptake value (SUV max) measures FDG activity in the primary tumor, and serves as a prognostic biomarker for lymph node status and illness outcomes [9,10].

The study purpose of this case was to assess the early therapeutic response in a 43-year-old patient with locally advanced cervical cancer (LACC) using both 18F-FDG PET/CT and MRI, as well as to investigate the relationship between follow-up imaging data and mutual correlation. We wanted to know if the post-treatment 18F-FDG PET/CT and MRI results were linked with progression-free and OS rates after concurrent chemoradiotherapy (CCRT). In addition, we looked at the effect of early and follow-up diagnostic imaging on future therapeutic management. Our results showed that 18F-FDG PET/CT outperformed MRI in the early assessment of treatment response in our patient after CCRT. Both imaging modalities offered data that could be used as predictive biomarkers of outcomes and survival in LACC patients.

## 2. Case Presentation

### 2.1. Clinical History

A 43-year-old female patient from an urban environment, with no personal pathological history, presented herself to the gynecology department of the Bihor County Emergency Clinical Hospital with vaginal bleeding. Affirmatively, the symptomatology started a month ago. Following the gynecological examination, a bleeding tumor formation on the cervix was described. At the biological level, no important changes were observed. The pathology management of the patient was performed by a multidisciplinary team of oncologists, surgeons, radiologists, gynecologists, and pathologists.

### 2.2. Imaging, Treatment Strategies, and Response

**Figure 1:**
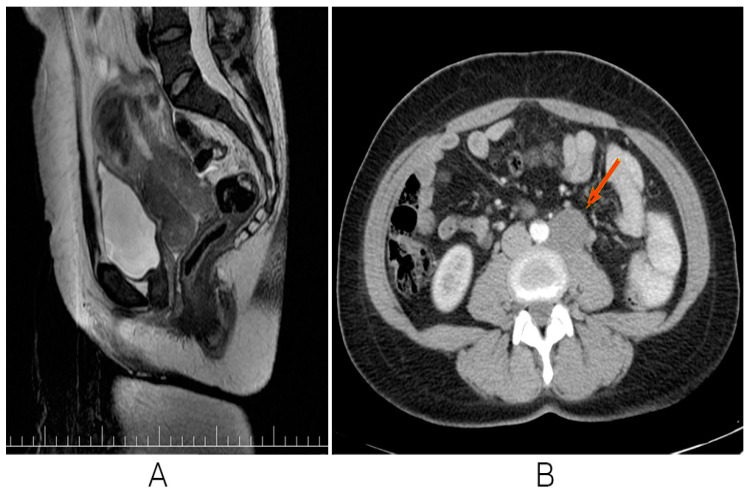
MRI SAG T2 sequence: cervical uterine hypointense lesion without gadolinium enhancement. (**A**) Contract CT. Arterial-phase retroperitoneal adenopathy (**B**).

An MRI of the pelvis was performed, which revealed a T2 hypointense lesion measuring 6.5 cm × 6 cm × 5 cm centered on the cervix, without gadolinium enhancement. External common iliac ganglia on the left side were also described. Other important mentions are two uterine body fibromas and bilateral ovarian cysts without any clinical relevance. An abdominal CT with contrast agent was performed, which designated retroperitoneal adenopathy with voluminous ganglion located on the inferior retroperitoneum measuring 3.5 cm × 3 cm. No other prevalent findings were recorded. All the manifestations of the observed results are mentioned in detail in Figure 1.

**Figure 2:**
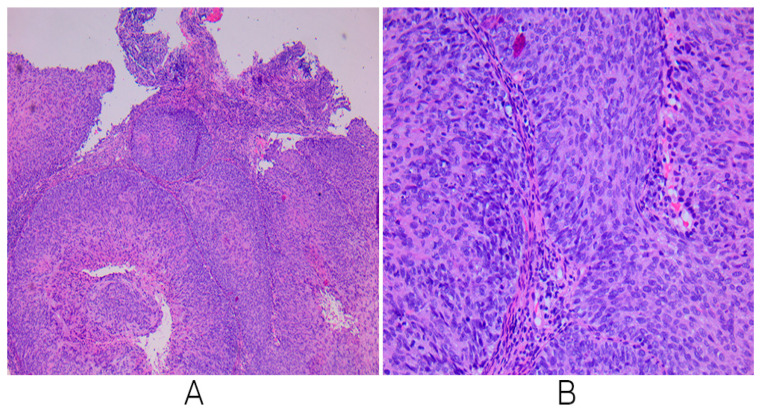
Microscopic examinations reveal a tumoral proliferation composed of nests of tumoral cells, separated by fine bands of connective tissue; the neoplastic cells are of medium size, irregular cellular birders, eosinophilic cytoplasm, and oversized hyperchromatic nuclei with moderate atypia, consistent with the diagnosis of a non-keratinized squamous cell carcinoma that is moderately differentiated (**A**): microscopic examination ×4, (**B**): microscopic examination ×20.

A cervical biopsy’s histological diagnosis is the gold standard for diagnosing cervical precancerous lesions and cervical cancer. After performing the biopsy from the cervix, the histopathological diagnosis of non-keratinized squamous carcinoma of the cervix G2 was confirmed (Figure 2).

**Figure 3:**
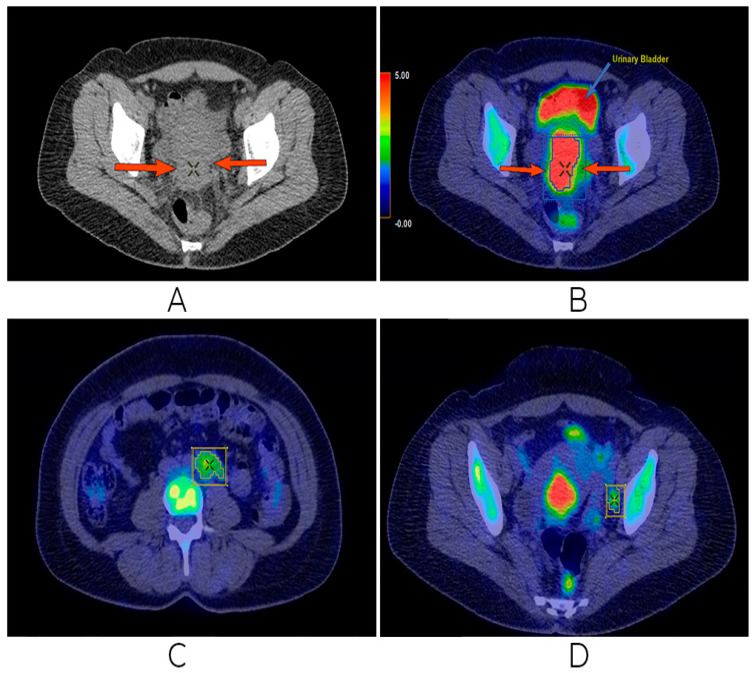
First FDG PET/CT examination. High FDG uptake in uterine cervical lesion (**A**,**B**), left para-aortic retroperitoneal (**C**), and left pelvic (external iliac) (**D**) adenopathies, both with FDG uptake.

An FDG PET-CT was performed, which revealed a large tumor measuring 6.5 cm × 6 cm × 5 cm centered on the cervix, with high FDG uptake (SUVlbm = 9.2). The tumor showed preserved rectal and vesical interfaces. The presence of common and external iliac adenopathy was confirmed, too. It is noted that the voluminous ganglion of 3.5 cm × 3 cm was located at the inferior retroperitoneal, left tangent to the bifurcation of the aorta, with FDG uptake at the periphery (SUVlbm = 3.9) and a necrotic central area. No other changes in pathology or FDG uptake were clearly seen (Figure 3).

**Figure 4:**
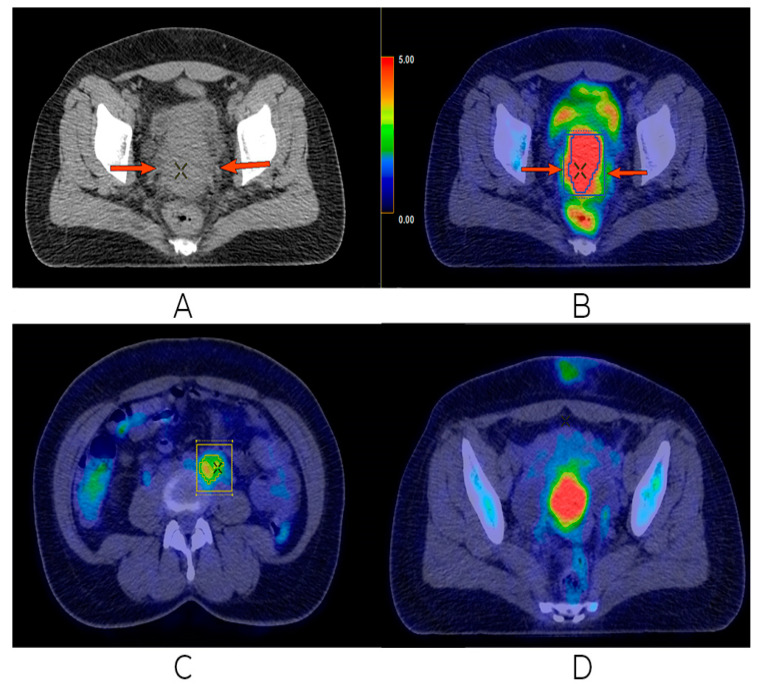
Second FDG PET/CT. Discrete progression in the uterine cervical tumor (**A**,**B**), as well as progression in the left aortic retroperitoneal adenopathy (**C**), but with remission of the left external iliac nodules (**D**).

According to the clinical, imaging, and histopathological examinations, a precise diagnosis of non-keratinizing squamous carcinoma of the uterine cervix FIGO stage III C1 was established. Clinically, the patient presented an ECOG performance status of 0, without biological changes. The multidisciplinary oncological team decided to start oncological treatment with neoadjuvant chemotherapy by using Carboplatin AUC 5 and Paclitaxel 175 mg/m^2^ every 3 weeks. Chemotherapy was well tolerated with the mention that, after the second administration of chemotherapy, the patient complained of peripheral paresthesia of the upper limbs, respectively, and complained of a slight pain in the hypogastrium. After the two cycles, an imaging assessment was performed with FDG PET-CT. Compared to the previous examination before the therapeutic outcome, the following is described. There were no changes in the size of the cervical tumor (7 cm × 6 cm), but with higher FDG uptake (current SUVlbm = 12.3 compared to previous SUVlbm = 9.2). The inferior retroperitoneal ganglion, left para-aortic, with dimensions of 3.8 cm × 3 cm with central necrosis, but with focal FDG uptake, was preserved (current SUVlbm = 5.1 compared to previous SUVlbm = 3.9). In the left external iliac, two nodes of 1.2 cm–1.3 cm can be distinguished, but without FDG uptake. Bilateral grade 2 hydronephrosis with preservation of renal function was observed. No new detectable or FDG uptake lesions were observed (Figure 4).

**Figure 5:**
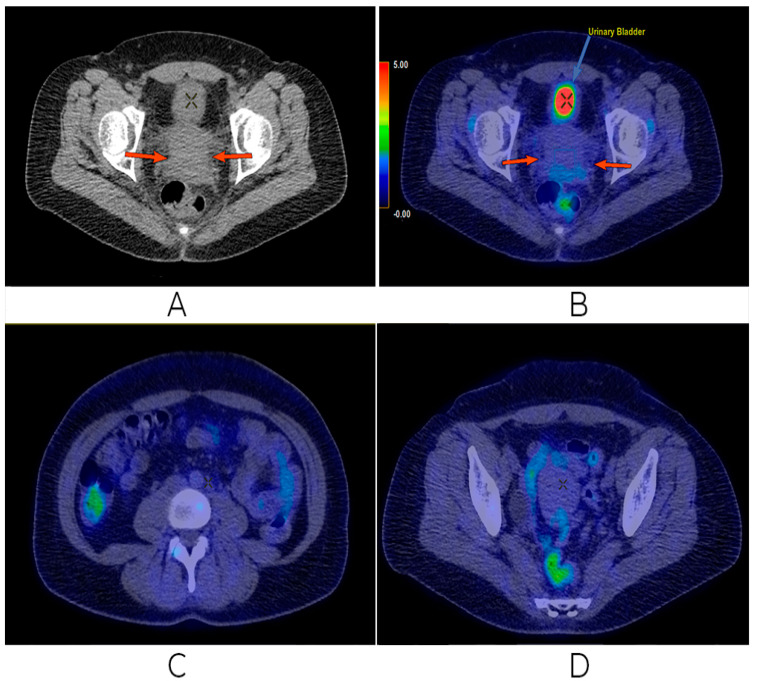
Last FDG PET/CT performed. Complete metabolic remission of the uterine cervical tumor (**A**,**B**), as well as the disappearance of the retroperitoneal (**C**) and left external iliac (**D**) adenopathies.

The treatment continued with conventional 3D external radiotherapy with a total dose of 63 Gy on the tumor volume, respectively, and chemotherapy for the purpose of radio sensitization. Chemotherapy was performed with a standard treatment dose of Cisplatin 100 mg/m^2^ every 3 weeks. Radiotherapy sessions were well tolerated, with the mention that the patient had mild nausea after taking Cisplatin. After the end of external radiotherapy, three more sessions of HDR brachytherapy with Ir^192^ with 7 Gy per session were performed every week. After the end of the local treatments, 4 weeks later, an imaging assessment was performed with PET CT with FDG. Compared to the previous two examinations, the evolution was favorable; the complete response and persistence of a residual mass of about 2.5 cm at the level of the cervix was observed, but without associated FDG capture. Grade 2 right hydronephrosis was in discrete remission, with the preservation of renal function. Almost complete morpho-metabolic remission of the retroperitoneal adenopathy mentioned in the previous examination was observed. No pelvic or inguinal lymphadenopathy, morphological changes, or FDG activity were detectable (Figure 5).

After the end of the treatment, the patient remained in the oncological record, following periodic imaging assessments with a CT of the chest, pelvis, and the abdomen without local and distant relapse. There were no tumor markers at any stage of disease evaluation. The post-therapeutic complications were represented by the vaginal stenosis installed 6 months after the end of the radiotherapeutic treatment. No vaginal dilator was used during the process of vaginal dilator installation.

## 3. Discussion

PET/CT is crucial for accurately delineating radiotherapy volumes, protecting the bone marrow of the patient from excessive radiation, and precise brachytherapy. Radiation use is always found to be an essential factor to cure women suffering from CC. The rapid advancements of radiotherapy now enable the delivery of high doses to tumor sites located near normal tissues while precisely sculpting the dose to spare the surrounding tissues. Although chemoradiotherapy (CRT) has improved survival rates, achieving optimal loco-regional control remains a significant challenge, emphasizing the need for supplementary treatment options. An enhanced understanding of the tumor microenvironment including factors like hypoxia and angiogenesis offers potential for incorporating advanced targeted molecular therapies. Progress in biological imaging modalities has substantially impacted the evaluation of treatment responses to new therapeutic regimens [11,12].

In our presented case, anatomical pictures were used to quantify and assess tumor extent and the biology of the tumor and the neighboring normal tissues. The merging of anatomical and biological pictures becomes critical and allows for the mapping of molecular distributions to guide external beam radiotherapy [13]. For instance, significant discrepancies in tumor volume have been observed between MRI and FDG-PET, likely because of considerable geometric modifications in the positions of the cervix, corpus uteri next to the bladder, and rectal filling [14,15].

The most widely used tracer in PET or PET/CT imaging is fluorine-18-labeled fluoro-2-deoxy-D-glucose (^18^F-FDG). Adaptive radiotherapy and chemotherapeutic treatment strategies accurately define disease status, which is critical and important to prevent the unnecessary irradiation of normal tissue. FDG-PET allows for the modification of radiation treatment volumes, enabling the safe delivery of accurate doses to FDG-positive lymph nodes. The SUV (standardized uptake value) is applied to PET imaging, typically utilizing the F18-2-fluoro-2-deoxy-D-glucose (FDG) to target the tumor. The SUV max (maximum standardized uptake value) measures the level of FDG activity in the primary tumor, serving as a prognostic biomarker for lymph node status and illness outcomes [10]. Additionally, the histology and differentiation of cervical cancer have been shown to affect FDG uptake. Follow-up or surveillance by using ^18^F-FDG PET/CT serves as a prognostic indicator for the overall survival of patients [16,17,18]. In cervical tumors, FDG uptake has been shown to vary based on histologic features and differentiation. Notably, squamous cell tumors exhibit significantly higher SUVmax values compared to non-squamous cell cancers, which aligns with our findings. This can be attributed to the tumor proliferation rates, reflecting tumor aggressiveness [5,19,20]. Our findings were also found to be consistent with follow-up observations in a patient with squamous cell carcinoma. Over a six-month period, this patient exhibited good survival rates, a decrease in SUVmax value (from 19.4 to 18), reduced tumor size, and stable FDG retention. Histological screening presents substantial potential for accurate prognosis and significantly reduces the incidence of metastatic cancer and associated mortality. After the start of RT, within 20 days, almost a 50% reduction in tumor volume was observed by a study performed by Lin et al. [19]. Tumor shrinkage level is a key factor in adjusting the dose and volume and in adaptive radiotherapy, and helps intensively to define the protocol for brachytherapy. Also, observed changes on the MRI, such as signal intensity and diffusion coefficient, can provide information regarding outcomes related CC after chemoradiotherapy. Our study showed that the FDG uptake variability was associated with the change in tumor size, and this can also be related to the factors discussed [21,22].

Women who actively engage in screening programs are more likely to be diagnosed at earlier stages of the disease [23,24]. After the treatment procedures were performed, the patient remained in the oncological record via periodic imaging assessments with a CT of the chest, abdomen, and pelvis, without local and distant relapse. In our study, the mean SUVmax was 6.71 (range of 3.3–10.6) among seven patients with various stages of cervical cancer. Conversely, a research study of 240 patients with various stages of cervical cancer by Kidd et al. reported a mean SUVmax of 11.62 (range of 2.50–50.39), which was significantly higher than our dataset. This discrepancy may be attributed to the comparatively larger sample size [10]. The post-therapeutic complications are represented by the vaginal stenosis installed 6 months after the end of the radiotherapeutic treatment. Locally advanced and metastatic illnesses are treated with systemic neoadjuvant chemotherapy to minimize the size of the original tumor and metastatic nodules before deciding on surgery. Consequently, it has been concluded that population-based screening programs in various countries facilitate the early detection of cervical cancer, thereby extending the survival of women diagnosed with metastatic stages of the disease [25]. Responsive primary tumors have been associated with increased rates of survival. Targeted therapy has emerged as a critical strategy in the treatment of many tumors [26]. However, its use in cervical cancer is currently limited in comparison to other types of cancer. One limitation of the present study is that more data and research of similar cases could explore the benefits in a better way. Nonetheless, ongoing research and clinical trials are investigating the beneficial effects of targeted therapies in cervical cancer treatment. Therapeutic approaches in cervical cancer must be carefully tailored to produce the best potential outcome for each patient.

## 4. Conclusions

It is strongly evident that ^18F^FDG-PET/CT plays an important role in the primary evaluation of cervical cancer, notably in determining lymph node status and diagnosing distant metastases, which contributes to accurate staging and influences therapeutic decisions. It has become popular for establishing tumor prognosis, measuring treatment response, evaluating disease recurrence, properly defining radiotherapy volumes, protecting active bone marrow from high radiation doses, and allowing for precise brachytherapy administration. Although CRT has improved survival rates, loco-regional control remains a substantial problem, emphasizing the need for additional treatments to increase efficacy. Advances in understanding the tumor microenvironment, hypoxia, and angiogenesis provide possibilities for applying new molecularly targeted medicines. Further innovations in the field of biological imaging are required, which may have a substantial impact on the assessment of treatment responses to the evolving therapeutic techniques.

## Data Availability

The original contributions presented in the study are included in the article; further inquiries can be directed to the corresponding authors.

## Abbreviations

FIGO	International Federation of Gynecology and Obstetrics
CCRT	concurrent chemoradiotherapy
PET	positron emission tomography
SCC	squamous cell carcinoma
CT	computed tomography
CRT	concurrent radiotherapy
FDG	18F-fluoro-D-glucose
MRI	Magnetic Resonance Imaging
SUV	standardized uptake value
SUVmax	maximum standardized uptake value
LACC	locally advanced cervical cancer
NACT	neoadjuvant chemotherapy
OS	overall survival
Ir^192^	Iridium-192
CC	cervical cancer

## References

[1] Cohen P.A., Jhingran A., Oaknin A., Denny L. (2019). Cervical cancer. Lancet.

[2] Pfaendler K.S., Tewari K.S. (2016). Changing paradigms in the systemic treatment of advanced cervical cancer. Am. J. Obstet. Gynecol..

[3] Peiretti M., Zapardiel I., Zanagnolo V., Landoni F., Morrow C.P., Maggioni A. (2012). Management of recurrent cervical cancer: A review of the literature. Surg. Oncol..

[4] Lima G.M., Matti A., Vara G., Dondi G., Naselli N., De Crescenzo E.M., Giuseppe A., Perrone A.M., De Iaco P., Nanni C. (2018). Prognostic value of posttreatment 18F-FDG PET/CT and predictors of metabolic response to therapy in patients with locally advanced cervical cancer treated with concomitant chemoradiation therapy: An analysis of intensity- and volume-based PET parameters. Eur. J. Nucl. Med. Mol. Imaging.

[5] Son H., Kositwattanarerk A., Hayes M.P. (2010). PET/CT evaluation of cervical cancer: Spectrum of disease. Radiographics.

[6] Park W., Park Y.J., Huh S.J. (2005). The usefulness of MRI and PET imaging for the detection of parametrial involvement and lymph node metastasis in patients with cervical cancer. Jpn. J. Clin. Oncol..

[7] Pecorelli S. (2009). Revised FIGO staging for carcinoma of the vulva, cervix, and endometrium. Int. J. Gynaecol. Obstet..

[8] Molnar O., Straciuc O.M., Mihutiu S., Lazar L. (2024). Impact of PET/CT Imaging with FDG in Locally Advanced Cervical Carcinoma- A Literature Review. Curr. Oncol..

[9] Kidd E.A., Siegel B.A., Dehdashti F., Grigsby P.W. (2007). The standardized uptake value for F-18 fluorodeoxyglucose is a sensitive predictive biomarker for cervical cancer treatment response and survival. Cancer.

[10] Kidd E.A., Spencer C.R., Huettner P.C., Siegel B.A., Dehdashti F., Rader J.S. (2009). Cervical cancer histology and tumor differentiation affect 18F-fluorodeoxyglucose uptake. Cancer.

[11] Azizah A.M., Hashimah B., Nirmal K., Siti Zubaidah A.R., Puteri N.A., Nabihah A., Sukumaran R., Balqis B., Nadia S.M.R., Sharifah S.S.S. (2019). Malaysia National Cancer Registry Report (MNCR) 2012–2016.

[12] Norsaadah B., Rampal K.G., Rahmah M.A., Naing N.N., Biswal B.M. (2011). Diagnosis delay of breast cancer and its associated factors in Malaysian women. BMC Cancer.

[13] Meechan G., Collins J., Petrie K.J. (2003). The relationship of symptoms and psychological factors to delay in seeking medical care for breast symptoms. Prev. Med..

[14] Ma D.J., Zhu J.M., Grigsby P.W. (2011). Tumor volume discrepancies between FDG-PET and MRI for cervical cancer. Radiother. Oncol..

[15] Viswanathan C., Silvana F., Patnana M., Sagebiel T., Iyer R.B. (2018). FDG-PET assessment of cervical cancer. PET Clin..

[16] Kitajima K., Suenaga Y., Ueno Y. (2014). Fusion of PET and MRI for staging of uterine cervical cancer: Comparison with contrast-enhanced ^18^F-FDG PET/CT and pelvic MRI. Clin. Imaging.

[17] Ponisio M.R., Fowler K.J., Dehdashti F. (2016). The Emerging Role of PET/MR Imaging in Gynecologic Cancers. PET Clin..

[18] Metser U., Golan O., Levine C.D., Even-Sapir E. (2005). Tumor lesion detection: When is integrated positron emission tomography/computed tomography more accurate than side-by-side interpretation of positron emission tomography and computed tomography?. J. Comput. Assist. Tomogr..

[19] Lin L.L., Yang Z., Mutic S., Miller T.R., Grigsby P.W. (2006). FDG-PET imaging for the assessment of physiologic volume response during radiotherapy in cervix cancer. Int. J. Radiat. Oncol. Biol. Phys..

[20] Dicu-Andreescu I., Marincaș A., Ungureanu V., Ionescu S., Prunoi V., Brătucu E., Simion L. (2023). Current Therapeutic Approaches in Cervical Cancer Based on the Stage of the Disease: Is There Room for Improvement?. Medicina.

[21] Ho J.C., Fang P., Cardenas C.E., Mohamed A.S.R., Fuller C.D., Allen P.K. (2019). Volumetric assessment of apparent diffusion coefficient predicts outcome following chemoradiation for cervical cancer. Radiother. Oncol. J. Eur. Soc. Ther. Radiol. Oncol..

[22] Liu B., Ma W.L., Zhang G.W., Sun Z., Zhong J.M., Wei M.Q. (2019). Changes in magnetic resonance T2-weighted imaging signal intensity correlate with concurrent chemoradiotherapy response in cervical cancer. J. Contemp. Brachyther..

[23] Taplin S.H., Ichikawa L., Buist D.S.M., Seger D., White E. (2004). Evaluating organized BC screening implementation: The prevention of late-stage disease?. Cancer Epidemiol. Prev. Biomark..

[24] Li M., Wang H., Qu N., Piao H., Zhu B. (2024). Breast cancer screening and early diagnosis in China: A systematic review and meta-analysis on 10.72 million women. BMC Womens Health.

[25] Gould H.J., Norleans J., Ward T.D., Reid C., Paul D. (2018). Selective lysis of breast carcinomas by simultaneous stimulation of sodium channels and blockade of sodium pumps. Oncotarget.

[26] Vora C., Gupta S. (2019). Targeted therapy in cervical cancer. ESMO Open.

