# Correction: Molnar et al. PET CT Imaging with FDG in the Therapeutical Management of Locally Advanced Cervical Cancer Diagnosed in a 43-Year-Old Patient: Case Report and Review of the Literature. *Biomedicines* 2025, *13*, 83

**DOI:** 10.3390/biomedicines14030676

**Published:** 2026-03-16

**Authors:** Ottó Molnar, Simona Mihuțiu, Oreste Mihai Straciuc, Alexandra Vesa, Liviu Lazar

**Affiliations:** 1 Doctoral Studies Department, Biomedical Science, 410087 Oradea, Romania; straciuc.mihai@gmail.com (O.M.S.); lazarlv@yahoo.com (L.L.); 2 Department of Medicine-Psycho-Neuroscience and Recovery, Faculty of Medicine and Pharmacy, 410073 Oradea, Romania; 3 Oncology Department, Pelican Hospital, 410469 Oradea, Romania; 4 Centrul PET/CT Pozitron Diagnosztika, 410035 Oradea, Romania; 5 Department of Morphological Sciences, Faculty of Medicine and Pharamacy, 410073 Oradea, Romania; alexvesa92@yahoo.com; 6 Băile Felix Medical Rehabilitation Hospital, 417500 Băile Felix, Romania

**References**

With this correction, references 23 and 24 have been replaced, as the original references cited incorrect articles due to human error during formatting [1]. The authors state that the scientific conclusions are unaffected. This correction was approved by the Academic Editor. The original publication has also been updated.

23. Taplin, S.H.; Ichikawa, L.; Buist, D.S.M.; Seger, D.; White, E. Evaluating organized BC screening implementation: The prevention of late-stage disease? *Cancer Epidemiol. Prev. Biomark.* **2004**, *13*, 225–234. https://doi.org/10.1158/1055-9965.EPI-03-0206.
24. Li, M.; Wang, H.; Qu, N.; Piao, H.; Zhu, B. Breast cancer screening and early diagnosis in China: A systematic review and meta-analysis on 10.72 million women. *BMC Womens Health* **2024**, *24*, 97. https://doi.org/10.1186/s12905-024-02924-4. PMID: 38321439; PMCID: PMC10848517.
